# Nasopharyngeal carriage, *spa* types and antibiotic susceptibility profiles of *Staphylococcus aureus* from healthy children less than 5 years in Eastern Uganda

**DOI:** 10.1186/s12879-019-4652-5

**Published:** 2019-12-02

**Authors:** David Patrick Kateete, Benon B. Asiimwe, Raymond Mayanja, Brian Mujuni, Freddie Bwanga, Christine F. Najjuka, Karin Källander, Elizeus Rutebemberwa

**Affiliations:** 1 Department of Immunology and Molecular Biology, Makerere University College of Health Sciences, Kampala, Uganda; 2 Department of Medical Microbiology, Makerere University College of Health Sciences, Kampala, Uganda; 3 Makerere University Walter Reed Project, Kampala, Uganda; 4 Malaria Consortium, London, UK; 5 Department of Public Health Sciences, Karolinska Institutet, Stockholm, Sweden; 6 Makerere University School of Public Health, Kampala, Uganda

**Keywords:** *Staphylococcus aureus*, Carriage, MSSA/MRSA, Urban/rural, Iganga/Mayuge districts, Multidrug resistant, Genotyping

## Abstract

**Background:**

*Staphylococcus aureus* carriage is a known risk factor for staphylococcal disease. However, the carriage rates vary by country, demographic group and profession. This study aimed to determine the *S. aureus* carriage rate in children in Eastern Uganda, and identify *S. aureus* lineages that cause infection in Uganda.

**Methods:**

Nasopharyngeal samples from 742 healthy children less than 5 years residing in the Iganga/Mayuge Health and Demographic Surveillance Site in Eastern Uganda were processed for isolation of *S. aureus*. Antibiotic susceptibility testing based on minimum inhibitory concentrations (MICs) was determined by the BD Phoenix™ system. Genotyping was performed by *spa* and SCC*mec* typing.

**Results:**

The processed samples yielded 144 *S. aureus* isolates (one per child) therefore, the *S. aureus* carriage rate in children was 19.4% (144/742). Thirty one percent (45/144) of the isolates were methicillin resistant (MRSA) yielding a carriage rate of 6.1% (45/742). All isolates were susceptible to rifampicin, vancomycin and linezolid. Moreover, all MRSA were susceptible to vancomycin, linezolid and clindamycin. Compared to methicillin susceptible *S. aureus* (MSSA) isolates (68.8%, 99/144), MRSA isolates were more resistant to non-beta-lactam antimicrobials –trimethoprim/sulfamethoxazole 73.3% (33/45) vs. 27.3% (27/99) [*p* < 0.0001]; erythromycin 75.6% (34/45) vs. 24.2% (24/99) [*p* < 0.0001]; chloramphenicol 60% (27/45) vs. 19.2% (19/99) [p < 0.0001]; gentamicin 55.6% (25/45) vs. 25.3% (25/99) [*p* = 0.0004]; and ciprofloxacin 35.6% (16/45) vs. 2% (2/99) [p < 0.0001]. Furthermore, 42 MRSA (93.3%) were multidrug resistant (MDR) and one exhibited high-level resistance to mupirocin. Overall, 61 MSSA (61.6%) were MDR, including three mupirocin and clindamycin resistant isolates. Seven *spa* types were detected among MRSA, of which t037 and t064 were predominant and associated with SCC*mec* types I and IV, respectively. Fourteen *spa* types were detected in MSSA which consisted mainly of t645 and t4353.

**Conclusions:**

*S. aureus* carriage rate in healthy children in Eastern Uganda is high and comparable to rates for hospitalized patients in Kampala. The detection of mupirocin resistance is worrying as it could rapidly increase if mupirocin is administered in a low-income setting. *S. aureus* strains of *spa* types t064, t037 (MRSA) and t645, t4353 (MSSA) are prevalent and could be responsible for majority of staphylococcal infections in Uganda.

## Background

A causal relationship between *Staphylococcus aureus* carriage and infection is well documented [1, 2]. The anterior nares and nasopharynx are the most important sites for *S. aureus* colonization in humans [3]. Generally, the prevalence of *S. aureus* nasal carriage ranges from 20 to 30% however, it varies by country, profession and demographic group [2, 4]. Notably, these estimates are from the developed countries and their applicability to African settings where infection control practices and surveillance for antimicrobial resistance are inadequate/nonexistent [5], is debatable. Furthermore, information on the prevalence, population structure and molecular epidemiology of *S. aureus* carriage in healthy individuals in Africa is scarce [6]. Although the prevalence and correlates of *S. aureus* nasal carriage in hospitalized adult patients and health workers in Uganda has been documented [7–9], carriage rates in the community especially among children who are more vulnerable to staphylococcal infections, is not known. Generally *S. aureus* studies in Africa have focused on clinical and/or nosocomial isolates, which limits our understanding of the *S. aureus* population structure on the continent [1].

The *Streptococcus pneumoniae*, *Haemophilus influenzae* and *Moraxella catarrhalis* nasopharyngeal carriage rates in healthy children in rural Eastern Uganda were found to be high at 58.6% (89/152), 15% (23/152) and 11% (16/152), respectively [10]. These organisms alongside *S. aureus*, are the main causes of bacterial pneumonia [10], the leading cause of death in children less than 5 years of age [11]. In a study by Rutebenderwa et al. [10], the antibiotic susceptibility profiles and carriage rates of *Streptococcus pneumoniae*, *Morexella catarrhalis* and *Haemophilus influenzae* in children less than 5 years residing in the Iganga/Mayuge Health and Demographic Surveillance Site (IMHDSS) in Eastern Uganda, was determined. However, the rates and characteristics of *S. aureus*, a frequent cause of infections in children, were not documented. Therefore, this follow-up study aimed to determine the carriage rate and antibiotic susceptibility patterns of *S. aureus* from children in Eastern Uganda. The study also aimed to identify *S. aureus* lineages that can cause infection in community and/or health care settings in Uganda. Due to frequent interaction between health care workers from Uganda’s only national referral hospital (Mulago in Kampala) and community members, we hypothesized that *S. aureus* isolates from the hospital and community will generally have the same genetic background.

## Methods

### Study setting and identification of *S. aureus*

This cross-sectional study was conducted between February and October 2011, nested in studies/projects that investigated the pneumococcal carriage rate in healthy children at the IMHDSS. The IMHDSS is located in Iganga and Mayuge districts in Eastern Uganda, towards the border between Uganda and Kenya. Prior to the current study on *S. aureus*, there was a pilot study in 2008 that investigated pneumococcal carriage in children at the IMHDSS [10]. Subsequently, a follow-up study was conducted in 2011, in which ~ 1300 children less than 5 years were screened for pneumococcal carriage [unpublished observations]. The 2011 follow-up study had several objectives one of which was investigating the integrated community case management of malaria and pneumonia in communities and appropriate treatment of pneumonia symptoms in children less than five years of age [12]. The 2011 study also investigated pneumococcal carriage in children at the IMHDSS [unpublished observations], and the current study on *S. aureus* was nested in this arm of the project, in which samples from 742 children (of ~ 1300 children investigated for pneumococcal carriage) were concurrently processed for isolation of *S. aureus*.

**Fig. 1:**
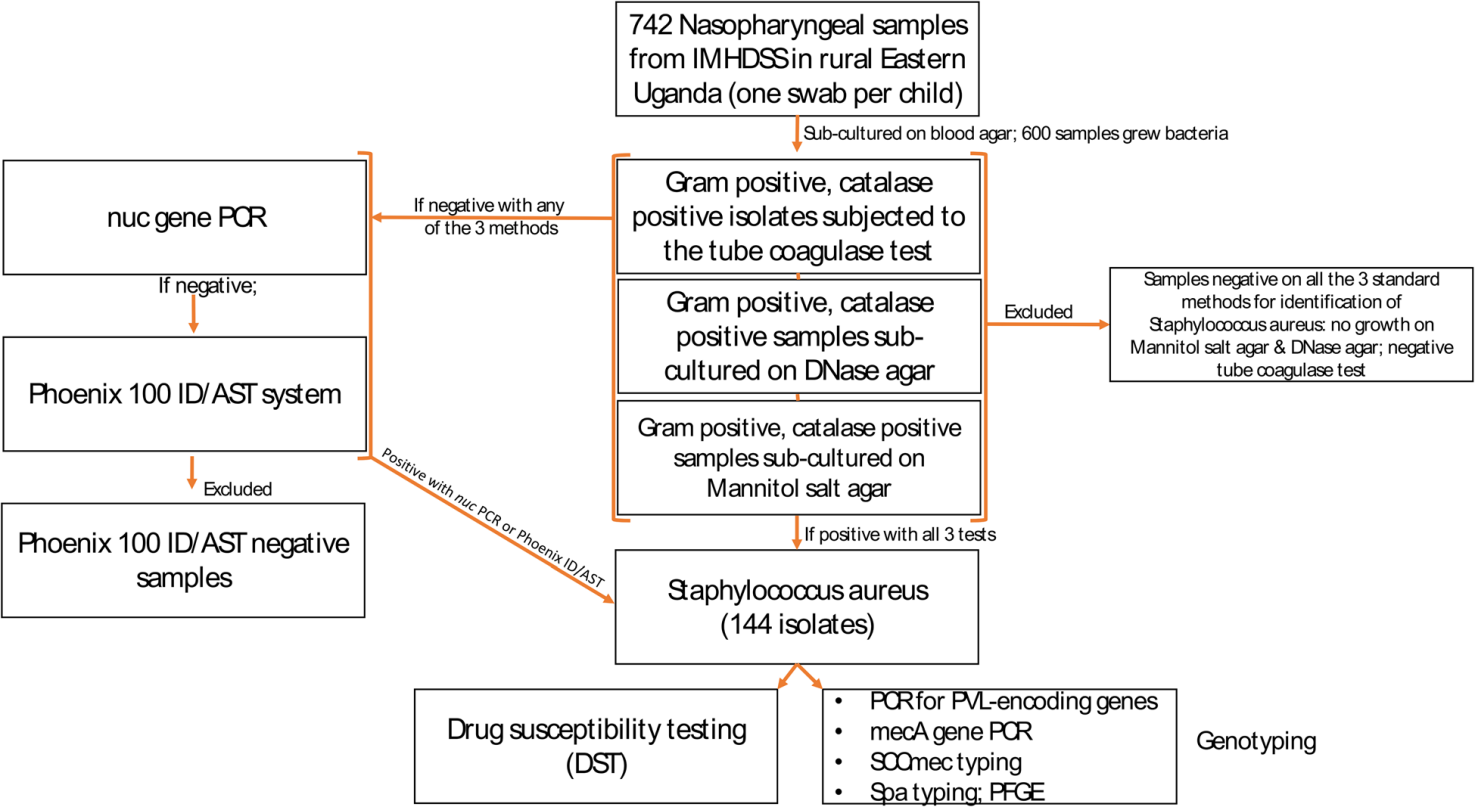
Study flow chart illustrating the procedure for the isolation and identification of *S. aureus*

The procedure for isolation and identification of *S. aureus* is depicted in Fig. 1. Briefly, presumptive *Staphylococcus* colonies were re-streaked on blood agar plates or on tryptic soy agar plates (for samples with no growth on blood agar) and incubated at 37 °C for 24 h in a CO_2_ incubator. To identify *S. aureus*, we subjected Gram-positive and catalase positive isolates to three different methods commonly used to identify *S. aureus*: (i) tube coagulase testing, (ii) growth on DNase agar, and (iii) growth on Mannitol salt agar [13]. Isolates that tested positive on all the three methods were considered to be *S. aureus*, otherwise isolates that tested negative with one or two methods were subjected to PCR-detection of the *nuc* gene [13]. Isolates were considered to be *S. aureus* if a 270 base pair fragment was identified on agarose gel electrophoresis. *Nuc* gene PCR-negative isolates were further evaluated with the BD Phoenix 100 ID/AST expert system for identification as previously described [14–16]. Isolates that tested negative with all the confirmatory tests for identification of *S. aureus* (i.e. tube coagulase test, *nuc* gene PCR and BD Phoenix 100 ID/AST) were excluded from further analysis.

### Antibiotic susceptibility testing

Antibiotic susceptibility testing was performed based on minimum inhibitory concentrations (MICs) using the BD Phoenix 100 ID/AST expert system. Multidrug resistance (MDR) was defined as isolates resistant to three or more classes of antimicrobials.

### Genotyping and other molecular procedures

To determine the *S. aureus* lineages, *spa* typing was performed as described previously [17]. Briefly, PCR products were purified with QIAquick PCR purification kit (Qiagen) and sequenced at MBN Laboratories (Kampala, Uganda) or ACGT Inc. (Wheeling, IL, USA) using forward and reverse primers used in the PCRs. To obtain *spa* types, sequences were submitted to an online spaTyper server http://spatyper.fortinbras.us/ and confirmed by cross-checking the repeats with the Ridom *Spa* Server http://spaserver2.ridom.de/spatypes.shtml To identify *S. aureus* lineages that are likely to cause infection in Uganda, we compared *spa* types for *S. aureus* at the IMHDSS to previously described *spa* types at Mulago Hospital in Kampala [9], as well as pastoral communities in Western Uganda [18, 19]. Further, SCC*mec* types among MRSA isolates were determined by using a previously published PCR protocol [20]. To detect the PVL-encoding genes (*lukS-PV* and *lukF-PV*), isolates were screened by PCR as previously described [8, 9]. For isolates with frequently occurring *spa* types, pulse field gel electrophoresis (PFGE) analysis was performed according to Asiimwe et al. [18]. Briefly, isolates were subjected to Sma I digestion using the CHEF Genomic DNA Plug kit (Bio-Rad) according to a standardized procedure [18], and PFGE run using a CHEF DRIII system (Bio-Rad). The InfoQuest FP (v5) software (Bio-Rad Laboratories) was used to analyze the PFGE profiles and interpreted according to Tenover et al. [21]. Cluster analysis was achieved using Dice similarity coefficients and the unweighted pair group method with averages (UPGMA) at 1.5% optimization and 1.5% position tolerance. Isolates displaying ≥95% similarity (~ 3 band difference) were assigned the same profile [21]. In all procedures reference strains ATCC-43300 -*mecA*+, PVL- (MRSA) and ATCC-29213 -mecA-, PVL- (MSSA) & ATCC-25923 -*mecA*-, PVL+ (MSSA) were used as positive or negative controls. Apart from *spa* typing in which all the PCR products were sequenced, DNA sequencing of amplified segments of *mecA* and PVL encoding genes was done for selected isolates and confirmed by BLAST-searching at NCBI https://blast.ncbi.nlm.nih.gov/Blast.cgi

Statistical analysis was performed with the SPSS statistical software (version 14.0, Chicago, IL, USA). A *p*-value less than 0.05 was considered statistically significant. We used the Chi Square test to determine the relationship between the frequencies of *spa* types, SCC*mec* types, and setting.

## Results

### MSSA and MRSA carriage rates

The processed samples yielded 600 Gram-positive and catalase positive isolates (one isolate per sample/child), of which 144 were confirmed to be *S. aureus* (one isolate per sample/child) (Fig. 1 and Additional file 1: Table S1 and Additional file 2: Table S2). Thus, the nasopharyngeal carriage rate of *S. aureus* in the children was 19.4% (144/742). Forty five (31.3%, 45/144) of the isolates were confirmed to be MRSA yielding a carriage rate of 6.1% (45/742). The MSSA carriage rate was 13.3% (99/742).

### Spa types, resistance patterns and antibiotypes

In this study, all the isolates were susceptible to rifampicin, vancomycin and linezolid, while all the MRSA were susceptible to vancomycin, linezolid and clindamycin (Additional file 1: Table S1 and Additional file 2: Table S2). Compared to MSSA isolates (68.8%, 99/144), MRSA isolates were more resistant to non-beta-lactam antimicrobials i.e. trimethoprim/sulfamethoxazole (co-trimoxazole) 73.3% (33/45) vs. 27.3% (27/99) [*p* < 0.0001], erythromycin 75.6% (34/45) vs. 24.2% (24/99) [p < 0.0001], chloramphenicol 60% (27/45) vs. 19.2% (19/99) [p < 0.0001], gentamicin 55.6% (25/45) vs. 25.3% (25/99) [*p* = 0.0004] and ciprofloxacin 35.6% (16/45) vs. 2% (2/99) [p < 0.0001]. One MRSA isolate (K2283) exhibited high-level mupirocin resistance (HLMup^r^) while 42 (93.3%, 42/45) were MDR including the mupirocin resistant isolate (Additional file 1: Table S1 and Additional file 2: Table S2). The proportion of PVL+ MSSA and PVL+ MRSA was 9/99 (9.1%) and 19/45 (13.2%), respectively. All PVL+ MRSA isolates were MDR and generally the presence of *PVL* genes was associated with the MDR phenotype (*P* = 0.0332). Seven *spa* types (t064, t4353, t002, t037, t355, t3092 and t12939) were detected among MRSA isolates, of which t064 (20%, 9/45) and t037 (15.6%, 7/45) were predominant (Additional file 1: Table S1 and Additional file 2: Table S2). *Spa* types t037 and t064 were significantly associated with MRSA and SCC*mec* types I & IV respectively, with t037 exclusively occurring in MRSA (Table 1). A general description of the number of clusters observed on PFGE analysis and diversity of the collection is shown in Additional file 3: Figure S1.

**Table 1:** Frequency of *spa* types among *S. aureus* isolates from children in IMHDSS, Eastern Uganda

*Spa* type	MDR (%)		MSSA		MRSA		Total		*P*-value
	Yes	No	Frequency	RF	Frequency	RF	Frequency	RF	
t064	10 (66.7)	05 (33.3)	06	6.1	9	20	15	10.4	= 0.0118
t645	06 (55.4)	05 (45.5)	11	11.1	0	0	11	7.6	= 0.0205
t4353	06 (60)	04 (40)	9	9.1	1	2.2	10	7	= 0.1324
t002	06 (85.7)	01 (14.3)	6	6.1	1	2.2	7	5	= 0.3158
t037	06 (85.7)	01 (14.3)	0	0	7	15.6	7	5	= 0.0001
t078	–	–	2	2	0	0	2	1.4	
t355	–	–	1	1	1	2.2	2	1.4	
t3092	–	–	1	1	1	2.2	2	1.4	
t12939	–	–	0	0	1	2.2	1	0.7	
t3662	–	–	1	1	0	0	1	0.7	
t318	–	–	1	1	0	0	1	0.7	
t1456	–	–	1	1	0	0	1	0.7	
t10394	–	–	1	1	0	0	1	0.7	
t1476	–	–	1	1	0	0	1	0.7	
t2168	–	–	1	1	0	0	1	0.7	
t213	–	–	1	1	0	0	1	0.7	
Unknown	–	–	4	–	0	0	4	–	
NT	–	–	17	–	4	–	21	–	
ND	–	–	35	–	20	–	55	–	
Total	–	–	99	–	45	–	144	–	

The predominant *spa* types are depicted in bold font. RF denotes Relative Frequency (%) i.e., the number of times that the event (i.e. *spa* lineage) occurred, divided by the total in that category

*NT* not type-able, *ND* not determined

Ninety nine (68.8%, 99/144) isolates were MSSA as they were cefoxtin susceptible and *mecA* negative (Additional file 1: Table S1 and Additional file 2: Table S2). The percentage and proportion of MSSA isolates resistant to penicillin, tetracycline, cotrimoxazole, erythromycin, gentamicin and chloramphenicol are 78.8% (78/99), 79.8% (79/99), 27.3% (27/99), 24.2% (24/99), 25.3% (25/99) and 19.2% (19/99), respectively. Three (3%, 3/99) MSSA isolates (K277–1, K251 and K1064) exhibited high-level mupirocin resistance and were also clindamycin resistant (Additional file 1: Table S1 and Additional file 2: Table S2). A total of 61 (61.6%, 61/99) MSSA were MDR including the three mupirocin resistant isolates (Additional file 1: Table S1 and Additional file 2: Table S2). Fourteen *spa* types were detected among MSSA, of which t645 (11.1%, 11/99), t4353 (9.1%, 9/99), t064 (6.1%, 6/99) and t002 (6.1%, 6/99) were predominant (Additional file 1: Table S1 and Additional file 2: Table S2). *Spa* type t645 exclusively occurred in MSSA (Table 1). There were other *spa* types that exclusively occurred in MSSA e.g. t078, t3662, t318, t1456, t10394, t1476, t2168 and t213 (Table 1) but their frequencies were too low (i.e. ≤2 occurrences) to allow meaningful analysis.

Table 2 summarizes the antibiotypes and their relationship with *spa* and SCC*mec* types. Overall, a total of 38 and 28 antibiotypes were detected among MSSA and MRSA isolates, respectively. The most prevalent antibiotypes in MSSA and MRSA had the resistance patterns PEN-TET (17.2%) and FOX-PEN-TET-SXT-ERY-CHL-GEN (15.6%), respectively (Table 2).

**Table 2:** Antibiotypes among MSSA and MRSA and their relationship with *spa* types

	Antibiotype	Resistance profile	No. isolates showing this pattern (%)	Major *spa* types (frequency)	SCC*mec* type (frequency)
MSSA	S1	PEN-TET	17 (17.2)	t064 (4), t4353 (3), t645 (2), t355 (1)	Not applicable
	S2	PEN-TET-ERY	11 (11.1)	t002 (3), t645 (1), t078 (1), t4353 (1), t2168 (1)	
	S3	PEN-TET-GEN	9 (9.1)	t318 (1), t213 (1), t1476 (1)	
	S4	PEN	6 (6.1)	t002 (1), t645 (1), t4353 (1)	
	S5	TET	6 (6.1)	t4353 (1)	
	S6	PEN-TET-CHL-GEN	4 (4.4)	t645 (1)	
	S7	PEN-TET-SXT	4 (4.4)	t002 (1), t1456 (1)	
	S8	PEN-TET-SXT-CHL	3 (3)	–	
	S9	PEN-SXT-ERY-CLI-MUP	3 (3)	–	
	S10	PEN-TET-CHL	2 (2)	t645 (1), t4353 (1)	
	S11	TET-SXT-CHL	2 (2)	t3662 (1), t10394 (1)	
	S12	PEN-TET-ERY-CHL	2 (2)	t064 (1), t3092 (1)	
	S13	PEN-SXT	2 (2)	t4353 (1)	
	S14	PEN-TET-SXT-ERY	2 (2)	t645 (1)	
	S38	- (Pan-susceptible)	3 (3)	t064 (1)	
MRSA	R1	PEN-FOX-TET-SXT-ERY-CHL-GEN	7 (15.6)	- (4), t064 (3)	I (4), IV (3)
	R2	PEN-FOX-TET-SXT-ERY-CHL-GEN-CIP	4 (9)	–	I (3), IV (1)
	R3	PEN-FOX-TET-SXT-ERY-CHL-GEN	4 (9)	–	I (3), IV (1)
	R4	PEN-PEN-TET-SXT-ERY	3 (6.7)	t002 (1), t064 (1)	I (1), II (1), IV (1)
	R5	PEN-FOX-TET-SXT-ERY-CIP	3 (6.7)	t064 (1)	IV (3)
	R6	PEN-FOX-TET-SXT-ERY-GEN	2 (4.4)	t064 (1)	I (1), IV (1)

Shown are antibiotypes depicted by two or more isolates. Antibiotypes depicted by only one isolate are shown in Additional file 1: Table S1

*FOX* cefoxitin, *PEN* penicillin, *TET* tetracycline, *SXT* trimethoprim/sulfamethoxazole or co-trimoxazole, *ERY* erythromycin, *CHL* chloramphenicol, *GEN* gentamicin, *CIP* ciprofloxacin, *CLI* clindamycin, *RIF* rifampicin, *MUP* Mupirocin High level, *VAN* vancomycin, *LZD* linezolid, *MSSA* Methicillin susceptible *S. aureus*, *MRSA* Methicillin resistant *S. aureus*

**Fig. 2:**
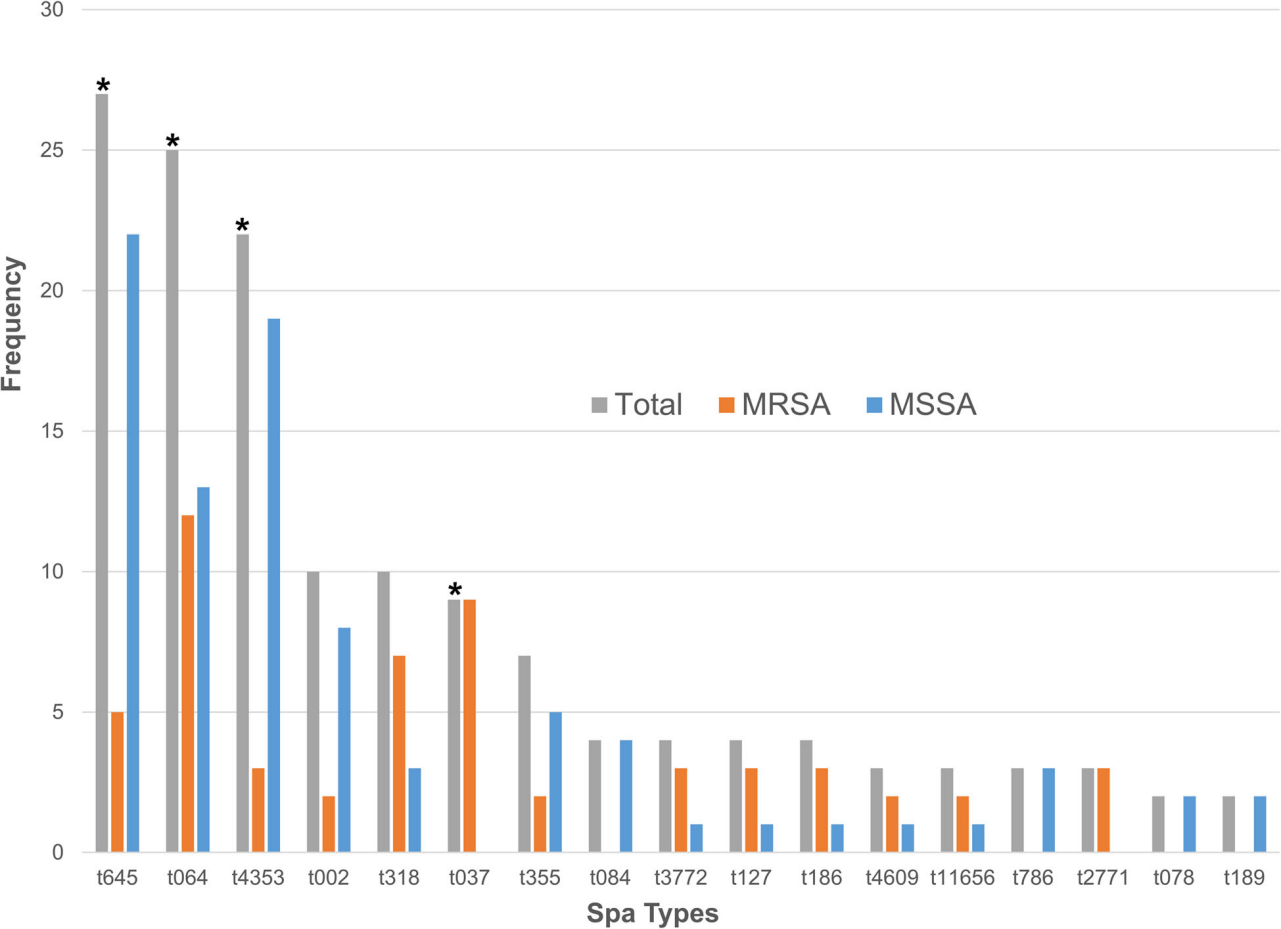
The most common *S. aureus spa* types identified in Uganda. Asterisks indicate frequencies for common *spa* types in Uganda and their association with either MRSA (t037, t064) or MSSA (t645, t4353)

When the genotypes of *S. aureus* isolates were compared with previously characterized isolates in Uganda, it was observed that the *spa* types detected at IMHDSS were previously reported from Mulago Hospital in Kampala, but slightly different from rural Western Uganda (Additional file 5: Figure S2). The number of isolates from the other sites (i.e. Mulago Hospital and rural Western Uganda) were as follows: 105 (Mulago Hospital, 64 & 41 from Seni et al. [9] & Kateete et al. [8], respectively) and 73 (rural Western Uganda) [19]. Of these, MRSA isolates were 113 (65 Mulago Hospital and 48 rural Western Uganda). Overall, 40 *spa* types accounted for MSSA/MRSA clones, of which t645, t064, t4353, t002, t318, t037, t355, t084, t3772, t127 and t186 were predominant (Fig. 2 and Additional file 4: Table S3). The frequent *spa* lineages in each of the sites were t064, t645, t4353, t002 and t037 (IMHDSS); t645, t4353, t064, t084, t355, t3772 and t4609 (Mulago Hospital); and t318, t064, t645, t186, t11656, t127, t786 and t2771 (rural Western Uganda) (Additional file 5: Figure S2). *Spa* type t037 exclusively occurred in MRSA and it was detected only at IMHDSS and Mulago Hospital. On the other hand, *spa* types t645 and t4353 occurred in all the three sites and they were significantly associated with MSSA (Additional file 4: Table S3 and Fig. 2). When isolates from the three sites were analyzed, *spa* types t4353, t002 and t355 were neither associated with MRSA or MSSA. Interestingly, *spa* type t064 that was significantly associated with MRSA at IMHDSS was not associated with MRSA at Mulago Hospital (Additional file 4: Table S3).

## Discussion

*Staphylococcus aureus* infection in Uganda was first reported at Mulago Hospital in1958 [22]. Since then, *S. aureus* has remained a leading cause of infection in Uganda and beyond [23–32], and it is second only to the pneumococcus among the frequent causes of pneumonia in children in Africa [1, 10, 11, 33]. Nasal/nasopharyngeal carriage of bacteria including *S. aureus* is a main risk factor for hospital- and community-acquired infections, as carriage provides a reservoir from which bacteria spread when there is breach of the body barriers (e.g. skin) or when the immune system is weakened. In this study, the *S. aureus* nasopharyngeal carriage rate (19.4%) in children in Eastern Uganda was found to be high but it falls within the reported estimates for children i.e. 18.12–38.5% [4]. The MRSA carriage rate (6.1%) was also high and comparable to estimates for adult patients at Mulago Hospital in Kampala [8, 9]. Further, the MRSA carriage rate was higher than the reported rates for children in developed countries (i.e. 0.8–1.5%) [4, 34] and developing countries (e.g. 2.3%, 14/622 for urban/rural school children in Gondar, Ethiopia [35]; 0.6%, 85/950 for adult inpatients in a Kenyan government hospital [36]). However, the study populations are diverse making direct comparisons difficult.

Several factors could be responsible for the high MRSA carriage rate in Ugandan children e.g. carriage by parents and previous exposure to antibiotics as one third of the children at the IMHDSS had prior antibiotic exposure particularly ampicillin and co-trimoxazole. Overcrowding is another factor that could have contributed to the observed high carriage rates in children as the pilot study at the IMHDSS found the median number of household members per bedroom (as a measure of crowding) to be four [10]. Large households of ≥5 members are associated with high *S. aureus* nasal carriage rates [1]. The demographic characteristics typical of the children sampled were described previously [10, 12]. Most children at the IMHDSS live in rural areas and their primary source of water is spring water and well water. By 2008, only 1% of the children had access to piped water and the median number of children per household is 2.

In this study, the proportion of MSSA isolates resistant to penicillin and tetracycline was 78–79.8%, while resistance to co-trimoxazole, erythromycin, chloramphenicol, gentamicin, clindamycin and ciprofloxacin was generally low. These findings are consistent with reports that penicillin resistance rates for *S. aureus* have remained in this range or higher since the late 1970s [37]. In fact, penicillin resistant *S. aureus* in Uganda was first reported at Mulago Hospital in Kampala in 1958 [22], at relatively lower prevalence (i.e. 28%, 27/98) compared to the rate reported in this study (91%). Furthermore, MRSA isolates in this study were MDR and this is also consistent with various reports on MRSA [33, 38]. However, it is important to note that the resistance profiles among countries would depend on the different practices of antibiotic use between the countries, antibiotic stewardship and enforcement of infection control practices. Overall, clindamycin, rifampicin, vancomycin and linezolid were the most effective antibiotics in this study, in contrast to findings from Ethiopia where gentamicin and ciprofloxacin were the most effective among isolates from primary school children [35].

Mupirocin is a relatively new drug that was introduced in Uganda in the 2000s [39]. It is used as a topical antibiotic to treat skin infections (e.g. impetigo, inflammation of hair follicles, minor skin infections, boils) caused by *S. aureus* and *Streptococcus pyogenes*. Mupirocin is also commonly used for nasal decolonization of MSSA and MRSA in patients and health care workers in the developed countries [5, 33, 37]. We have reported a high rate (4%) of mupirocin resistance (high-level) and this is consistent with the reported rates for this drug in Africa (5–50%) [40]. The detection of mupirocin resistance in Uganda is worrying as MRSA decolonization is generally not a common practice in this country. As mupirocin use in decolonization regimens leads to rapid emergence of resistance [33], its frequent use in Uganda for eradication of *Staphylococcus* colonization will likely spike an increase in resistance [40].

It is interesting to note that attempts to type staphylococci in Uganda date back to 1958 when phage typing was used to characterize *S. aureus* isolates at Mulago Hospital [22]. However, the typing of MRSA in Uganda was first reported in 1992 [41] (by multi-locus enzyme electrophoresis). Overall, this study has shown that *S. aureus* strains of *spa* types t645, t064, t4353, t002, t318, t037, t355, t084, t3772 and t127 are prevalent in Uganda, and they appear be common in Africa [36, 42, 43]. Consistent with this, a systematic review of the global distribution of *spa* types revealed that t064 and t037 are the most prevalent in Africa with t037 being exclusively associated with MRSA isolates [44]. The exception is t4353, which appears to be common in Uganda and other settings in East Africa [9, 19, 36] but rare in other African countries [42, 45]. Consequently, *S. aureus* infections in Uganda are likely to be associated with strains of *spa* types t064 & t037 (MRSA) and/or t645, t4353, t002 & t318 (MSSA/MRSA). *Spa* types t037 and t064 are frequently associated with MLST sequence types ST-30 & ST-8, respectively, while t645 is associated with ST-121 [46]. It is important to note that, while *spa* type t037 is frequently associated with hospital-associated MRSA strains, it has mainly been associated with SCC*mec* type III [47, 48] though other investigators have associated it with SCC*mec* types I & IVc [47].

There were certain limitations in this study. First, we used the http://spatyper.fortinbras.us/ server for identification of *spa* types as it is freely accessible but it is not the official database for determining *spa* types. However, several investigators [45, 49–51] have used the http://spatyper.fortinbras.us/ server to accurately determine *spa* types. Generally, PCR genotyping approaches are prone to errors in interpretation and the molecular marker itself can yield results that may depict homoplasy events and/or convergent evolution. This, and the fact that our sample size for MRSA isolates was relatively small, will require larger studies with robust approaches such as whole genome sequencing. Second, we were not able to type all the isolates recovered from the children. Because of this, and the fact that few studies have performed *spa* typing of *S. aureus* isolates in Uganda, the reported frequencies and associations should be taken with caution as frequencies could change with genotyping of more isolates. We also lacked information on clonal complexes based on MLST. Although it was inappropriate to relate isolates from children and adults, our aim was to describe the population structure of these isolates without drawing inferences on epidemiological linkages. Third, we used a cross-sectional design with a single sampling point to classify children as carriers implying that some children classified as non-carriers might have been intermittent carriers [34]. Furthermore, atypical *S. aureus* isolates were detected e.g. coagulase positive isolates that were *nuc* negative, and these could have been other species of coagulase positive staphylococci (e.g. *S. argenteus* [52]) misidentified as *S. aureus*. However, there is no single phenotypic test that can always accurately identify *S. aureus* and the detection of atypical isolates of *S. aureus* in this setting was reported before [13]. The Expert Identification Systems such as Phoenix ID/100 and Vitek2 GP Card (bioMérieux) may also not differentiate atypical *S. aureus* [52]. As such, we regarded *nuc* negative isolates to be *S. aureus* because the Phoenix ID/100 system used as a tie-breaker confirmed their identity.

## Conclusions

The nasopharyngeal carriage rate of *S. aureus* (MSSA/MRSA) in children in Eastern Uganda is high and comparable to estimates for adult patients at Mulago Hospital in Kampala. As high levels of MDR isolates were detected, outpatient treatment of *S. aureus* infections in children in Eastern Uganda might be difficult. Moreover, the detection of mupirocin resistant isolates is a cause for concern in that once mupirocin is introduced for *Staphylococcus* decolonization, it will likely spike a rapid increase in resistance in a low-income setting. Surveys of adult populations show a reduction in *S. aureus* nasal carriage rate and this is attributed to factors like improved personal hygiene, smaller families [1]. Hence, health education of target populations on standard hygiene practices is necessary for MRSA control in Uganda. Lastly, *S. aureus* isolates of *spa* types t037 & t064 (MRSA), and t645 & t4353 (MSSA) are prevalent in Uganda and could be responsible for majority of staphylococcal infections in the country.

## Supplementary information

**Additional file 1: Table S1.** *Spa* types and antibiotic susceptibility profiles of MSSA and MRSA from children less than 5 years in rural eastern Uganda.

**Additional file 2: Table S2.** General characteristics of *S. aureus* from children less than 5 years of age at the IMHDSS, eastern Uganda.

**Additional file 3: Figure S1.** PFGE analysis of *S. aureus* showing 10 clusters (A-J) of isolates with similar profiles hence genetically related. This analysis confirmed clusters (C & D) with MRSA isolates of *spa* type t064. +, PVL-positive; −, PVL-negative.

**Additional file 4: Table S3.** Frequency of *spa* types among *S. aureus* in Uganda.

**Additional file 5: Figure S2.** The most frequent *spa* types among *S. aureus* from the IMHDSS (panel A), Mulago Hospital (panel B) and rural western Uganda (panel C). Asterisks indicate frequencies for common *spa* types in Uganda and their association with either MRSA or MSSA. This analysis showed that MRSA infections in Uganda are more likely to be associated with *spa* types t064, t037, t645 and t318.

## Data Availability

All data generated or analyzed during this study are included in this published article [and its supplementary information files].

## Abbreviations

HLMup^r^: High-level mupirocin resistance
IMHDSS: Iganga-Mayuge Health & Demographic Surveillance Site
MIC: Minimum inhibitory concentrations
MLST: Multi-locus sequence typing
MRSA: Methicillin resistant *Staphylococcus aureus*
MSSA: Methicillin susceptible *Staphylococcus aureus*
NCBI: National Center for Biotechnology Information
PCR: Polymerase chain reaction
PVL: Panton Valentine leukocidin
SCC*mec*: Staphylococcal cassette chromosome mec
*Spa*: Staphylococcal protein A
SXT: Trimethoprim/sulfamethoxazole

## References

[1] Wertheim HF, Melles DC, Vos MC, van Leeuwen W, van Belkum A, Verbrugh HA, Nouwen JL (2005). The role of nasal carriage in Staphylococcus aureus infections. Lancet Infect Dis.

[2] de Benito S, Alou L, Becerro-de-Bengoa-Vallejo R, Losa-Iglesias ME, Gómez-Lus ML, Collado L, Sevillano D (2018). Prevalence of Staphylococcus spp. nasal colonization among doctors of podiatric medicine and associated risk factors in Spain. Antimicrob Resist Infect Control.

[3] Kates AE, Thapaliya D, Smith TC, Chorazy ML (2018). Prevalence and molecular characterization of Staphylococcus aureus from human stool samples. Antimicrob Resist Infect Control.

[4] Lucia Preoţescu L, Streinu-Cercel O (2013). Prevalence of nasal carriage of S aureus in children. Germs.

[5] Williams PCM, Isaacs D, Berkley JA (2018). Antimicrobial resistance among children in sub-Saharan Africa. Lancet Infect Dis.

[6] Abdulgader SMAA. Nasopharyngeal carriage with *Staphylococcus aureus* in healthy children during the first year of life-the Drakenstein child health study: PhD thesis, University of Cape Town; 2016.

[7] Bebell LM, Ayebare A, Boum Y, Siedner MJ, Bazira J, Schiff SJ, Metlay JP, Bangsberg DR, Ttendo S, Firth PG (2017). Prevalence and correlates of MRSA and MSSA nasal carriage at a Ugandan regional referral hospital. J Antimicrob Chemother.

[8] Kateete DP, Namazzi S, Okee M, Okeng A, Baluku H, Musisi NL, Katabazi FA, Joloba ML, Ssentongo R, Najjuka FC (2011). High prevalence of methicillin resistant Staphylococcus aureus in the surgical units of Mulago hospital in Kampala, Uganda. BMC Res Notes.

[9] Seni J, Bwanga F, Najjuka CF, Makobore P, Okee M, Mshana SE, Kidenya BR, Joloba ML, Kateete DP (2013). Molecular Characterization of Staphylococcus aureus from Patients with Surgical Site Infections at Mulago Hospital in Kampala, Uganda. PLOS ONE.

[10] Rutebemberwa E, Mpeka B, Pariyo G, Peterson S, Mworozi E, Bwanga F, Källander K (2015). High prevalence of antibiotic resistance in nasopharyngeal bacterial isolates from healthy children in rural Uganda: a cross-sectional study. Ups J Med Sci.

[11] Wardlaw T, Salama P, Johansson EW, Mason E (2006). Pneumonia: the leading killer of children. Lancet.

[12] Kalyango JN, Alfven T, Peterson S, Mugenyi K, Karamagi C, Rutebemberwa E (2013). Integrated community case management of malaria and pneumonia increases prompt and appropriate treatment for pneumonia symptoms in children under five years in eastern Uganda. Malar J.

[13] Kateete DP, Kimani CN, Katabazi FA, Okeng A, Okee MS, Nanteza A, Joloba ML, Najjuka FC (2010). Identification of Staphylococcus aureus: DNase and Mannitol salt agar improve the efficiency of the tube coagulase test. Ann Clin Microbiol Antimicrob.

[14] Carroll KC, Borek AP, Burger C, Glanz B, Bhally H, Henciak S, Flayhart DC (2006). Evaluation of the BD Phoenix automated microbiology system for identification and antimicrobial susceptibility testing of staphylococci and enterococci. J Clin Microbiol.

[15] Laboratory Procedures: BD Phoenix™ PMIC/ID Panels BPPP, BD Phoenix™ PID Panels [http://www.bd.com/]. Accessed 2011.

[16] Kateete DP, Kabugo U, Baluku H, Nyakarahuka L, Kyobe S, Okee M, Najjuka CF, Joloba ML (2013). Prevalence and antimicrobial susceptibility patterns of bacteria from milkmen and cows with clinical mastitis in and around Kampala, Uganda. PLoS One.

[17] Harmsen D, Claus H, Witte W, Rothganger J, Turnwald D, Vogel U (2003). Typing of methicillin-resistant Staphylococcus aureus in a university hospital setting by using novel software for spa repeat determination and database management. J Clin Microbiol.

[18] Asiimwe BB, Baldan R, Trovato A, Cirillo DM (2017). Prevalence and molecular characteristics of Staphylococcus aureus, including methicillin resistant strains, isolated from bulk can milk and raw milk products in pastoral communities of south-West Uganda. BMC Infect Dis.

[19] Asiimwe BB, Baldan R, Trovato A, Cirillo DM (2017). Molecular epidemiology of Panton-valentine Leukocidin-positive community-acquired methicillin resistant Staphylococcus aureus isolates in pastoral communities of rural south western Uganda. BMC Infect Dis.

[20] Boye K, Bartels MD, Andersen IS, Moller JA, Westh H (2007). A new multiplex PCR for easy screening of methicillin-resistant Staphylococcus aureus SCCmec types I-V. Clin Microbiol Infect.

[21] Tenover FC, Arbeit RD, Goering RV, Mickelsen PA, Murray BE, Persing DH, Swaminathan B (1995). Interpreting chromosomal DNA restriction patterns produced by pulsed-field gel electrophoresis: criteria for bacterial strain typing. J Clin Microbiol.

[22] Hennessey RS, Miles RA (1958). Staphylococcus aureus type 80 and human infections in Uganda. Br Med J.

[23] Herrmann M, Abdullah S, Alabi A, Alonso P, Friedrich AW, Fuhr G, Germann A, Kern WV, Kremsner PG, Mandomando I (2013). Staphylococcal disease in Africa: another neglected 'tropical' disease. Future Microbiol.

[24] Jacob ST, Moore CC, Banura P, Pinkerton R, Meya D, Opendi P, Reynolds SJ, Kenya-Mugisha N, Mayanja-Kizza H, Scheld WM (2009). Severe Sepsis in two Ugandan hospitals: a prospective observational study of management and outcomes in a predominantly HIV-1 infected population. PLoS One.

[25] Mugalu J, Nakakeeto MK, Kiguli S, Kaddu-Mulindwa DH (2006). Aetiology, risk factors and immediate outcome of bacteriologically confirmed neonatal septicaemia in Mulago hospital, Uganda. Afr Health Sci.

[26] Ojulong J, Mwambu T, Joloba M, Bwanga F, Kaddu-Mulindwa D (2009). Relative prevalence of methicilline resistant Staphylococcus aureus and its susceptibility pattern in mulago hospital, Kampala, Uganda. Tanzan J Health Res.

[27] Schaumburg F, Alabi AS, Peters G, Becker K (2014). New epidemiology of Staphylococcus aureus infection in Africa. Clin Microbiol Infect.

[28] Seni J, Najjuka CF, Kateete DP, Makobore P, Joloba ML, Kajumbula H, Kapesa A, Bwanga F (2013). Antimicrobial resistance in hospitalized surgical patients: a silently emerging public health concern in Uganda. BMC Res Notes.

[29] Anguzu JR, Olila D (2007). Drug sensitivity patterns of bacterial isolates from septic post-operative wounds in a regional referral hospital in Uganda. Afr Health Sci.

[30] Kizito M, Mworozi E, Ndugwa C, Serjeant GR (2007). Bacteraemia in homozygous sickle cell disease in Africa: is pneumococcal prophylaxis justified?. Arch Dis Child.

[31] Bachou H, Tylleskar T, Kaddu-Mulindwa DH, Tumwine JK (2006). Bacteraemia among severely malnourished children infected and uninfected with the human immunodeficiency virus-1 in Kampala, Uganda. BMC Infect Dis.

[32] Falagas ME, Karageorgopoulos DE, Leptidis J, Korbila IP (2013). MRSA in Africa: filling the global map of antimicrobial resistance. PLoS One.

[33] David MZ, Daum RS (2010). Community-associated methicillin-resistant Staphylococcus aureus: epidemiology and clinical consequences of an emerging epidemic. Clin Microbiol Rev.

[34] Kuehnert MJ, Kruszon-Moran D, Hill HA, McQuillan G, McAllister SK, Fosheim G, McDougal LK, Chaitram J, Jensen B, Fridkin SK (2006). Prevalence of Staphylococcus aureus nasal colonization in the United States, 2001–2002. J Infect Dis.

[35] Tigabu A, Tiruneh M, Mekonnen F (2018). Nasal Carriage Rate, Antimicrobial Susceptibility Pattern, and Associated Factors of Staphylococcus aureus with Special Emphasis on MRSA among Urban and Rural Elementary School Children in Gondar, Northwest Ethiopia: A Comparative Cross-Sectional Study. Adv Prev Med.

[36] Aiken AM, Mutuku IM, Sabat AJ, Akkerboom V, Mwangi J, Scott JAG, Morpeth SC, Friedrich AW, Grundmann H (2014). Carriage of Staphylococcus aureus in Thika level 5 hospital, Kenya: a cross-sectional study. Antimicrob Resist Infect Control.

[37] Gnanamani Arumugam, Hariharan Periasamy, Paul-Satyaseela Maneesh (2017). Staphylococcus aureus: Overview of Bacteriology, Clinical Diseases, Epidemiology, Antibiotic Resistance and Therapeutic Approach. Frontiers in Staphylococcus aureus.

[38] Kong EF, Johnson JK, Jabra-Rizk MA (2016). Community-associated methicillin-resistant Staphylococcus aureus: an enemy amidst us. PLoS Pathog.

[39] Mpairwe Y, Wamala S (2015). Antibiotic resistance in Uganda: situation analysis and recommendations.

[40] Shittu AO, Kaba M, Abdulgader SM, Ajao YO, Abiola MO, Olatimehin AO (2018). Mupirocin-resistant Staphylococcus aureus in Africa: a systematic review and meta-analysis. Antimicrob Resist Infect Control.

[41] Musser JM, Kapur V (1992). Clonal analysis of methicillin-resistant Staphylococcus aureus strains from intercontinental sources: association of the mec gene with divergent phylogenetic lineages implies dissemination by horizontal transfer and recombination. J Clin Microbiol.

[42] Eibach Daniel, Nagel Michael, Hogan Benedikt, Azuure Clinton, Krumkamp Ralf, Dekker Denise, Gajdiss Mike, Brunke Melanie, Sarpong Nimako, Owusu-Dabo Ellis, May Jürgen (2017). Nasal Carriage of Staphylococcus aureus among Children in the Ashanti Region of Ghana. PLOS ONE.

[43] Moremi N, Mshana SE, Kamugisha E, Kataraihya J, Tappe D, Vogel U, Lyamuya EF, Claus H (2012). Predominance of methicillin resistant Staphylococcus aureus-ST88 and new ST1797 causing wound infection and abscesses. J Infect Dev Ctries.

[44] Asadollahi P, Farahani NN, Mirzaii M, Khoramrooz SS, van Belkum A, Asadollahi K, Dadashi M, Darban-Sarokhalil D (2018). Distribution of the Most Prevalent Spa Types among Clinical Isolates of Methicillin-Resistant and -Susceptible *Staphylococcus aureus* around the World: A Review. Front Microbiol.

[45] Donkor ES, Jamrozy D, Mills RO, Dankwah T, Amoo PK, Egyir B, Badoe EV, Twasam J, Bentley SD (2018). A genomic infection control study for Staphylococcus aureus in two Ghanaian hospitals. Infect Drug Resist.

[46] Rao Q, Shang W, Hu X, Rao X (2015). Staphylococcus aureus ST121: a globally disseminated hypervirulent clone. J Med Microbiol.

[47] Mohammadi S, Sekawi Z, Monjezi A, Maleki M-H, Soroush S, Sadeghifard N, Pakzad I, Azizi-Jalilian F, Emaneini M, Asadollahi K (2014). Emergence of SCCmec type III with variable antimicrobial resistance profiles and spa types among methicillin-resistant Staphylococcus aureus isolated from healthcare- and community-acquired infections in the west of Iran. Int J Infect Dis.

[48] Neela V, Ghasemzadeh Moghaddam H, van Belkum A, Horst-Kreft D, Mariana NS, Ghaznavi Rad E (2009). First report on methicillin-resistant Staphylococcus aureus of Spa type T037, sequence type 239, SCCmec type III/IIIA in Malaysia. Eur J Clin Microbiol Infect Dis.

[49] Khan S, Sung K, Iram S, Nawaz M, Xu J, Marasa B (2016). Draft genome sequences of two methicillin-resistant clinical Staphylococcus aureus isolates. Genome Announc.

[50] Marasa BS, Khan S, Iram S, Sung K, Xu J (2015). Draft genome sequence of methicillin-resistant clinical Staphylococcus aureus isolate 51S (sequence type 291). Genome Announc.

[51] Seidl K, Leimer N, Palheiros Marques M, Furrer A, Holzmann-Bürgel A, Senn G, Zbinden R, Zinkernagel AS (2015). Clonality and antimicrobial susceptibility of methicillin-resistant Staphylococcus aureus at the university hospital Zurich, Switzerland between 2012 and 2014. Ann Clin Microbiol Antimicrob.

[52] Tong SYC, Schaumburg F, Ellington MJ, Corander J, Pichon B, Leendertz F, Bentley SD, Parkhill J, Holt DC, Peters G (2015). Novel staphylococcal species that form part of a Staphylococcus aureus-related complex: the non-pigmented Staphylococcus argenteus sp. nov. and the non-human primate-associated Staphylococcus schweitzeri sp. nov. Int J Syst Evol Microbiol.

