# Supplementary material for: Nasopharyngeal carriage, spa types and antibiotic susceptibility profiles of Staphylococcus aureus from healthy children less than 5 years in Eastern Uganda
Source: BMC Infect Dis. 2019 Dec 2;19:1023. doi: 10.1186/s12879-019-4652-5 (PMC6889221; doi:10.1186/s12879-019-4652-5)
Supplement: Supplementary file 1 — Additional file 1: Table S1. Spa types and antibiotic susceptibility profiles of MSSA and MRSA from children less than 5 years in rural eastern Uganda. [file 12879_2019_4652_MOESM1_ESM.docx]

**Table S1: Spa Types and antibiotic susceptibility profiles of MSSA & MRSA from children ≤5 years in rural eastern Uganda**

| **Isolate #** | ***PVL*** | ***mecA*** | **SCC*mec*** | **FOX** | **PEN** | **TET** | **SXT** | **ERY** | **CHL** | **GEN** | **CIP** | **CLI** | **MUP** | **RIF** | **VAN** | **LZD** | **MDR** | **Spa type** |
| --- | --- | --- | --- | --- | --- | --- | --- | --- | --- | --- | --- | --- | --- | --- | --- | --- | --- | --- |
| MSSA (n=99) | | | | | | | | | | | | | | | | | | |
| 72 | - | - | NA | S | **R** | **R** | S | **R** | S | S | S | S | S | S | S | S | **Yes** | **t002** |
| 124 | + | - | NA | S | **R** | **R** | S | S | S | S | **R** | S | S | S | S | S | **Yes** | ND |
| 128 | - | - | NA | S | **R** | **R** | S | S | S | S | S | S | S | S | S | S | No | NT |
| 136 | - | - | NA | S | **R** | **R** | S | S | S | S | S | S | S | S | S | S | No | **t645** |
| 142 | - | - | NA | S | **R** | **R** | S | S | S | S | S | S | S | S | S | S | No | **t645** |
| 160 | - | - | NA | S | **R** | **R** | **R** | S | S | S | S | S | S | S | S | S | **Yes** | **t002** |
| 182 | - | - | NA | S | **R** | **R** | S | S | **R** | S | S | S | S | S | S | S | **Yes** | **t645** |
| 213 | - | - | NA | S | **R** | **R** | S | **R** | S | S | S | S | S | S | S | S | **Yes** | **t002** |
| 353 | - | - | NA | S | S | **R** | **R** | S | **R** | S | S | S | S | S | S | S | **Yes** | t3662 |
| 993 | - | - | NA | S | **R** | **R** | S | S | S | **R** | S | S | S | S | S | S | **Yes** | t318 |
| 1243 | - | - | NA | S | **R** | S | S | S | S | S | S | S | S | S | S | S | No | **t002** |
| 3050 | - | - | NA | S | **R** | **R** | S | **R** | S | S | S | S | S | S | S | S | **Yes** | NT |
| 206B | - | - | NA | S | **R** | **R** | S | S | **R** | **R** | S | S | S | S | S | S | **Yes** | NT |
| 298C | - | - | NA | S | **R** | S | S | S | S | S | S | S | S | S | S | S | No | NT |
| 302A | - | - | NA | S | **R** | **R** | S | S | S | S | S | S | S | S | S | S | No | NT |
| K1000 | - | - | NA | S | **R** | S | S | S | S | S | S | S | S | S | S | S | No | **t4353** |
| K1018 | - | - | NA | S | S | **R** | S | S | S | S | S | S | S | S | S | S | No | **t4353** |
| K1026 | - | - | NA | S | S | **R** | S | S | **R** | **R** | S | S | S | S | S | S | **Yes** | **t645** |
| K1036 | - | - | NA | S | S | **R** | S | S | S | S | S | S | S | S | S | S | No | Unknown |
| K1044 | - | - | NA | S | **R** | **R** | S | **R** | **R** | **R** | S | S | S | S | S | S | **Yes** | **t4353** |
| K1083 | - | - | NA | S | **R** | **R** | S | **R** | S | S | S | S | S | S | S | S | **Yes** | **t645** |
| K1104 | - | - | NA | S | **R** | **R** | S | **R** | **R** | S | S | S | S | S | S | S | **Yes** | Unknown |
| K11076 | + | - | NA | S | S | **R** | S | S | S | S | S | S | S | S | S | S | No | ND |
| K1107C | - | - | NA | S | S | **R** | S | S | S | S | S | S | S | S | S | S | No | NT |
| K1113 | - | - | NA | S | **R** | S | S | S | S | S | S | S | S | S | S | S | No | ND |
| K1301 | - | - | NA | S | **R** | **R** | **R** | S | S | S | S | S | S | S | S | S | **Yes** | ND |
| K1357 | - | - | NA | S | **R** | **R** | S | **R** | S | S | S | S | S | S | S | S | **Yes** | NT |
| K137 | - | - | NA | S | **R** | **R** | **R** | S | S | S | S | S | S | S | S | S | **Yes** | NT |
| K163 | - | - | NA | S | **R** | **R** | **R** | **R** | **R** | S | S | S | S | S | S | S | **Yes** | ND |
| K224 | - | - | NA | S | **R** | S | **R** | S | S | S | S | S | S | S | S | S | No | **t4353** |
| K229B | - | - | NA | S | **R** | **R** | S | S | S | **R** | S | S | S | S | S | S | **Yes** | ND |
| K251 | - | - | NA | S | **R** | **R** | S | S | S | S | S | S | S | S | S | S | **Yes** | **t4353** |
| K260 | - | - | NA | S | **R** | **R** | **R** | S | S | S | S | S | S | S | S | S | **Yes** | t1456 |
| **Isolate #** | ***PVL*** | ***mecA*** | **SCC*mec*** | **FOX** | **PEN** | **TET** | **SXT** | **ERY** | **CHL** | **GEN** | **CIP** | **CLI** | **MUP** | **RIF** | **VAN** | **LZD** | **MDR** | **Spa type** |
| K268 | - | - | NA | S | **R** | **R** | S | **R** | S | S | S | S | S | S | S | S | **Yes** | **t4353** |
| K277 | - | - | NA | S | **R** | **R** | S | S | **R** | S | S | S | S | S | S | S | **Yes** | **t4353** |
| K281 | - | - | NA | S | **R** | S | **R** | **R** | I | S | S | S | S | S | S | S | **Yes** | NT |
| K282 | - | - | NA | S | **R** | **R** | S | S | S | **R** | S | S | S | S | S | S | No | ND |
| K287 | - | - | NA | S | **R** | **R** | S | S | S | S | S | S | S | S | S | S | **Yes** | **t4353** |
| K294 | - | - | NA | S | **R** | S | S | S | S | **R** | S | S | S | S | S | S | **Yes** | NT |
| K296 | - | - | NA | S | **R** | **R** | **R** | **R** | S | **R** | S | S | S | S | S | S | **Yes** | **t002** |
| K301 | - | - | NA | S | **R** | **R** | S | S | S | S | S | S | S | S | S | S | **Yes** | **t4353** |
| K312 | - | - | NA | S | **R** | **R** | S | S | S | S | S | S | S | S | S | S | **Yes** | NT |
| K32 | - | - | NA | S | **R** | **R** | S | S | S | S | S | S | S | S | S | S | **Yes** | **t064** |
| K330 | - | - | NA | S | **R** | **R** | S | S | S | **R** | S | S | S | S | S | S | **Yes** | ND |
| K34 | - | - | NA | S | S | S | I | S | S | **R** | S | S | S | S | S | S | No | ND |
| K341 | - | - | NA | S | **R** | **R** | S | S | S | **R** | S | S | S | S | S | S | **Yes** | ND |
| K35 | - | - | NA | S | S | **R** | S | S | S | S | S | S | S | S | S | S | No | ND |
| K351 | - | - | NA | S | **R** | **R** | S | S | S | **R** | S | S | S | S | S | S | **Yes** | ND |
| K37A | - | - | NA | S | **R** | S | S | S | I | S | S | S | S | S | S | S | No | ND |
| K40 | - | - | NA | S | **R** | S | S | S | S | S | S | S | S | S | S | S | No | NT |
| K418B | - | - | NA | S | **R** | **R** | S | S | S | S | S | S | S | S | S | S | No | ND |
| K421 | - | - | NA | S | S | **R** | S | S | S | S | S | S | S | S | S | S | No | ND |
| K422 | - | - | NA | S | **R** | **R** | S | **R** | S | S | S | S | S | S | S | S | **Yes** | Unknown |
| K429 | - | - | NA | S | **R** | **R** | **R** | S | **R** | S | S | S | S | S | S | S | **Yes** | NT |
| K445 | - | - | NA | S | **R** | **R** | **R** | **R** | S | S | S | S | S | S | S | S | **Yes** | ND |
| K50 | - | - | NA | S | S | **R** | S | S | S | **R** | S | S | S | S | S | S | No | ND |
| K510B | - | - | NA | S | **R** | **R** | S | **R** | S | S | S | S | S | S | S | S | **Yes** | t078 |
| K524 | - | - | NA | S | **R** | **R** | **R** | S | **R** | S | S | S | S | S | S | S | **Yes** | NT |
| K541 | - | - | NA | S | **R** | **R** | S | S | **R** | **R** | S | S | S | S | S | S | **Yes** | NT |
| K614 | + | - | NA | S | **R** | **R** | **R** | S | S | **R** | S | S | S | S | S | S | **Yes** | ND |
| K628C | - | - | NA | S | S | **R** | **R** | S | S | S | S | S | S | S | S | S | No | ND |
| K631 | - | - | NA | S | S | **R** | **R** | S | S | **R** | **R** | S | S | S | S | S | **Yes** | **t645** |
| K652 | + | - | NA | S | S | **R** | **R** | S | **R** | **R** | I | S | S | S | S | S | **Yes** | ND |
| K6520 | - | - | NA | S | S | **R** | **R** | S | S | S | S | S | S | S | S | S | No | **t645** |
| K663 | - | - | NA | S | S | **R** | **R** | S | S | **R** | S | S | S | S | S | S | **Yes** | t078 |
| K664C | - | - | NA | S | S | **R** | **R** | S | **R** | S | S | S | S | S | S | S | **Yes** | t10394 |
| K685C | - | - | NA | S | S | **R** | **R** | **R** | S | **R** | S | S | S | S | S | S | **Yes** | ND |
| K693 | - | - | NA | S | **R** | **R** | S | S | S | **R** | S | S | S | S | S | S | **Yes** | t1476 |
| K741 | - | - | NA | S | **R** | **R** | S | S | S | S | S | S | S | S | S | S | No | t355 |
| K87 | - | - | NA | S | **R** | **R** | S | S | S | **R** | S | S | S | S | S | S | **Yes** | ND |
| **Isolate #** | ***PVL*** | ***mecA*** | **SCC*mec*** | **FOX** | **PEN** | **TET** | **SXT** | **ERY** | **CHL** | **GEN** | **CIP** | **CLI** | **MUP** | **RIF** | **VAN** | **LZD** | **MDR** | **Spa type** |
| K901 | - | - | NA | S | **R** | S | S | S | S | S | S | S | S | S | S | S | No | **t645** |
| K931 | - | - | NA | S | S | **R** | S | S | **R** | S | S | S | S | S | S | S | No | ND |
| K992 | - | - | NA | S | **R** | **R** | S | S | S | S | S | S | S | S | S | S | No | ND |
| NR0810 | - | - | NA | S | **R** | **R** | S | **R** | S | S | S | S | S | S | S | S | Yes | t2168 |
| R01 | - | - | NA | S | **R** | **R** | S | S | S | S | S | S | S | S | S | S | No | **t064** |
| R010 | - | - | NA | S | **R** | **R** | S | S | **R** | **R** | S | S | S | S | S | S | No | ND |
| R03 | + | - | NA | S | **R** | S | **R** | S | S | S | S | S | S | S | S | S | No | NT |
| R04 | + | - | NA | S | **R** | **R** | **R** | **R** | S | S | S | S | S | S | S | S | Yes | **t645** |
| R05 | - | - | NA | S | **R** | **R** | S | S | S | S | S | S | S | S | S | S | No | ND |
| R07 | - | - | NA | S | **R** | **R** | S | S | S | S | S | I | S | S | S | S | No | ND |
| R09 | - | - | NA | S | **R** | I | **R** | S | S | **R** | S | S | S | S | S | S | **Yes** | ND |
| R11 | + | - | NA | S | **R** | **R** | S | **R** | S | S | S | S | S | S | S | S | **Yes** | ND |
| R12 | - | - | NA | S | **R** | **R** | S | **R** | **R** | S | S | S | S | S | S | S | **Yes** | t3092 |
| R13 | + | - | NA | S | **R** | **R** | S | S | **R** | **R** | S | S | S | S | S | S | **Yes** | **t645** |
| R15 | - | - | NA | S | **R** | **R** | S | S | S | S | S | S | S | S | S | S | No | **t064** |
| R16 | - | - | NA | S | S | S | S | S | S | S | S | S | S | S | S | S | No | ND |
| R180 | - | - | NA | S | S | S | S | S | S | S | S | S | S | S | S | S | No | NT |
| R190 | - | - | NA | S | **R** | **R** | S | S | S | S | I | S | S | S | S | S | No | ND |
| R200 | - | - | NA | S | **R** | **R** | S | **R** | S | S | S | S | S | S | S | S | **Yes** | **t002** |
| R21 | - | - | NA | S | **R** | **R** | **R** | S | **R** | S | S | S | S | S | S | S | **Yes** | Unknown |
| R22A | + | - | NA | S | **R** | S | S | S | S | S | S | S | S | S | S | S | No | **t645** |
| R23 | - | - | NA | S | **R** | **R** | S | S | S | S | S | S | S | S | S | S | No | **t064** |
| R25 | - | - | NA | S | **R** | **R** | S | **R** | **R** | S | S | S | S | S | S | S | **Yes** | **t064** |
| R26B | - | - | NA | S | **R** | **R** | S | S | S | S | S | S | S | S | S | S | No | ND |
| R28D | - | - | NA | S | **R** | **R** | S | S | S | **R** | S | S | S | S | S | S | **Yes** | t213 |
| R716 | - | - | NA | S | S | S | S | S | S | S | S | S | S | S | S | S | No | **t064** |
| K277-1 | - | - | NA | S | **R** | I | **R** | **R** | S | S | I | **R** | **R** | S | S | S | **Yes** | ND |
| K251 | - | - | NA | S | **R** | I | **R** | **R** | S | S | I | **R** | **R** | S | S | S | **Yes** | ND |
| K1064 | - | - | NA | S | **R** | I | **R** | **R** | S | S | I | **R** | **R** | S | S | S | **Yes** | ND |
| Total +/R MSSA (%) | 09 (9.1) | 0 (0) | NA | 0 (0) | 78 (78.8) | 79 (79.8) | 27 (27.3) | 24 (24.2) | 19 (19.2) | 25 (25.3) | 02 (2) | 03 (3) | 03 (3) | 0 (0) | 0 (0) | 0 (0) | 61 (61.6) |  |
| MRSA (n=45) | | | | | | | | | | | | | | | | | | |
| 52-1 | + | + | I | **R** | **R** | **R** | **R** | **R** | **R** | **R** | S | S | S | S | S | S | **Yes** | ND |
| R14 | - | + | I | **R** | **R** | **R** | **R** | S | S | S | S | S | S | S | S | S | **Yes** | **t037** |
| 1320-1 | - | + | I | **R** | **R** | **R** | **R** | **R** | **R** | **R** | **R** | S | S | S | S | S | **Yes** | ND |
| 244C-1 | - | + | I | **R** | **R** | **R** | **R** | **R** | **R** | **R** | S | S | S | S | S | S | **Yes** | ND |
| **Isolate #** | ***PVL*** | ***mecA*** | **SCC*mec*** | **FOX** | **PEN** | **TET** | **SXT** | **ERY** | **CHL** | **GEN** | **CIP** | **CLI** | **MUP** | **RIF** | **VAN** | **LZD** | **MDR** | **Spa type** |
| K1057-1 | - | + | I | **R** | **R** | **R** | S | S | **R** | **R** | S | S | S | S | S | S | **Yes** | **t037** |
| K2283 | - | + | I | **R** | **R** | S | S | S | S | S | **R** | S | **R** | S | S | S | **Yes** | **t037** |
| K264-1 | + | + | I | **R** | **R** | **R** | S | S | **R** | **R** | S | S | S | S | S | S | **Yes** | ND |
| K284-1 | - | + | I | **R** | **R** | **R** | **R** | **R** | **R** | **R** | S | S | S | S | S | S | **Yes** | ND |
| K36-1 | - | + | I | **R** | **R** | **R** | **R** | **R** | **R** | S | S | S | S | S | S | S | **Yes** | **t037** |
| K39-1 | - | + | I | **R** | **R** | **R** | **R** | **R** | **R** | **R** | **R** | S | S | S | S | S | **Yes** | ND |
| K4834-1 | - | + | I | **R** | **R** | **R** | S | S | **R** | **R** | S | S | S | S | S | S | **Yes** | **t037** |
| K370-1 | - | + | I | **R** | **R** | **R** | **R** | **R** | **R** | **R** | S | S | S | S | S | S | **Yes** | ND |
| K970-1 | - | + | I | **R** | **R** | **R** | I | S | S | S | I | S | S | S | S | S | No | **t037** |
| R030-1 | - | + | I | **R** | **R** | **R** | **R** | **R** | **R** | **R** | **R** | S | S | S | S | S | **Yes** | ND |
| R10-1 | - | + | I | **R** | **R** | **R** | **R** | **R** | **R** | S | **R** | S | S | S | S | S | **Yes** | t12939 |
| R110-1 | + | + | I | **R** | **R** | **R** | **R** | **R** | S | S | S | S | S | S | S | S | **Yes** | NT |
| R19-1 | - | + | I | **R** | **R** | **R** | **R** | **R** | **R** | **R** | S | S | S | S | S | S | **Yes** | ND |
| R220-1 | - | + | I | **R** | **R** | **R** | **R** | **R** | S | **R** | S | S | S | S | S | S | **Yes** | NT |
| 1322-1 | + | + | II | **R** | **R** | **R** | **R** | **R** | **R** | **R** | I | I | S | S | S | S | **Yes** | ND |
| 306C-1 | - | + | II | **R** | **R** | **R** | **R** | **R** | I | **R** | S | S | S | S | S | S | **Yes** | ND |
| K911-1 | - | + | II | **R** | **R** | **R** | S | **R** | **R** | **R** | **R** | S | S | S | S | S | **Yes** | ND |
| R160-1 | - | + | II | **R** | **R** | **R** | **R** | **R** | S | S | S | S | S | S | S | S | **Yes** | t002 |
| R17-1 | - | + | III | **R** | **R** | **R** | **R** | S | S | I | S | S | S | S | S | S | **Yes** | **t037** |
| 1325-1 | - | + | IV | **R** | **R** | **R** | **R** | **R** | **R** | I | S | S | S | S | S | S | **Yes** | t355 |
| 1326-1 | + | + | IV | **R** | **R** | **R** | S | **R** | S | S | **R** | S | S | S | S | S | **Yes** | ND |
| K2241 | - | + | IV | **R** | **R** | S | S | S | S | S | **R** | I | S | S | S | S | No | **t4353** |
| K2810-1 | - | + | IV | **R** | **R** | **R** | **R** | **R** | S | S | **R** | S | S | S | S | S | **Yes** | ND |
| R31B-1 | - | + | IV | **R** | **R** | **R** | **R** | **R** | S | **R** | **R** | S | S | S | S | S | **Yes** | **t064** |
| R310-1 | - | + | IV | **R** | **R** | **R** | **R** | **R** | **R** | **R** | S | S | S | S | S | S | **Yes** | **t064** |
| R33-1 | + | + | IV | **R** | **R** | **R** | **R** | **R** | **R** | **R** | S | S | S | S | S | S | **Yes** | **t064** |
| K3700-1 | - | + | IV | **R** | **R** | **R** | S | S | **R** | **R** | S | S | S | S | S | S | **Yes** | ND |
| R0100-1 | - | + | IV | **R** | **R** | **R** | **R** | **R** | S | S | S | S | S | S | S | S | **Yes** | **t064** |
| R020-1 | - | + | IV | **R** | **R** | S | **R** | S | **R** | **R** | S | S | S | S | S | S | **Yes** | NT |
| R0121 | - | + | IV | **R** | **R** | **R** | **R** | **R** | **R** | S | **R** | S | S | S | S | S | **Yes** | t3092 |
| R0300-1 | - | + | IV | **R** | **R** | **R** | **R** | **R** | **R** | S | S | S | S | S | S | S | **Yes** | ND |
| R06-1 | - | + | IV | **R** | **R** | **R** | **R** | **R** | S | **R** | S | S | S | S | S | S | **Yes** | **t064** |
| R08-1 | + | + | IV | **R** | **R** | **R** | **R** | **R** | **R** | **R** | S | S | S | S | S | S | **Yes** | **t064** |
| R18-1 | - | + | IV | **R** | **R** | **R** | S | **R** | **R** | S | **R** | S | S | S | S | S | **Yes** | ND |
| R20-1 | - | + | IV | **R** | **R** | I | **R** | **R** | **R** | **R** | S | S | S | S | S | S | **Yes** | **t064** |
| R27 | + | + | IV | **R** | **R** | **R** | S | **R** | S | S | S | S | S | S | S | S | No | **t064** |
| R26A-1 | - | + | IV | **R** | **R** | **R** | **R** | **R** | S | S | **R** | S | S | S | S | S | **Yes** | **t064** |
| **Isolate #** | ***PVL*** | ***mecA*** | **SCC*mec*** | **FOX** | **PEN** | **TET** | **SXT** | **ERY** | **CHL** | **GEN** | **CIP** | **CLI** | **MUP** | **RIF** | **VAN** | **LZD** | **MDR** | **Spa type** |
| K350C-1 | + | + | V | **R** | **R** | **R** | S | S | S | S | **R** | S | S | S | S | S | **Yes** | NT |
| K38-1 | - | + | V | **R** | **R** | **R** | **R** | **R** | **R** | **R** | I | S | S | S | S | S | **Yes** | ND |
| K60-1 | + | + | V | **R** | **R** | **R** | **R** | **R** | S | S | **R** | S | S | S | S | S | **Yes** | ND |
| R040-1 | - | + | V | **R** | **R** | **R** | **R** | **R** | **R** | **R** | **R** | S | S | S | S | S | **Yes** | ND |
| Total +/R MRSA (%) | 10 (22.2) | 45 (100) |  | 45 (100) | 45 (100) | 41 (91.1) | 33 (73.3) | 34 (75.6) | 27 (60) | 25 (55.6) | 16 (35.6) | 0 (0) | 01 (2.2) | 0 (0) | 0 (0) | 0 (0) | 42 (93.3) |  |
| **Grand Total +/R (%)** | **19 (13.2)** | **45 (31.3)** |  | **45 (31.3)** | **122 (84.7)** | **120 (83.3)** | **60 (41.7)** | **58 (40.3)** | **46 (32)** | **50 (34.7)** | **18 (12.5)** | **03 (2.1)** | **04 (2.8)** | **0 (0)** | **0 (0)** | **0 (0)** | **103 (71.5)** |  |

- FOX, cefoxitin; PEN, penicillin; TET, tetracycline; SXT, trimethoprim/sulfamethoxazole or co-trimoxazole; ERY, erythromycin, CHL, chloramphenicol; GEN, gentamycin; CIP, ciprofloxacin; CLI, clindamycin; RIF, rifampicin; MUP, Mupirocin High level; VAN, vancomycin; LZD, linezolid; MDR, multidrug resistant –resistance to three or more classes of antimicrobials; MSSA, Methicillin susceptible *S. aureus*; MRSA, Methicillin resistant *S. aureus***;** +, Positive; -, Negative; NT, Not typable; NA, Not applicable; ND, Not determined. The frequently occurring spa types are presented in bold-face font.
