# Supplementary material for: Nasopharyngeal carriage, spa types and antibiotic susceptibility profiles of Staphylococcus aureus from healthy children less than 5 years in Eastern Uganda
Source: BMC Infect Dis. 2019 Dec 2;19:1023. doi: 10.1186/s12879-019-4652-5 (PMC6889221; doi:10.1186/s12879-019-4652-5)
Supplement: Supplementary file 4 — Additional file 4: Table S3. Frequency of spa types among S. aureus in Uganda. [file 12879_2019_4652_MOESM4_ESM.docx]

**Table S3: Distribution of *spa* types among *S. aureus* in Uganda (n=172)**

| ***spa* type** | **MSSA** | | **MRSA** | | **Total** | | **P-value** |
| --- | --- | --- | --- | --- | --- | --- | --- |
|  | **Frequency** | **RF** | **Frequency** | **RF** | **Frequency** | **RF** |  |
| **t645** | 22 | 22.2 | 5 | 6.8 | 27 | 15.7 | **P = 0.0062** |
| **t064** | 13 | 13.1 | 12 | 16.4 | 25 | 14.5 | P = 0.5447 |
| **t4353** | 19 | 19.2 | 3 | 4.1 | 22 | 12.8 | **P = 0.0035** |
| **t002** | 8 | 8.1 | 2 | 2.7 | 10 | 5.8 | P = 0.1357 |
| **t318** | 3 | 3 | 7 | 9.6 | 10 | 5.8 | P = 0.0680 |
| **t037** | 0 | 0 | 9 | 12.3 | 9 | 5.2 | **P = 0.0004** |
| **t355** | 5 | 5.1 | 2 | 2.7 | 7 | 4.1 | P = 0.4331 |
| **t084** | 4 | 4 | 0 | 0 | 4 | 2.3 | P = 0.0847 |
| **t3772** | 1 | 1 | 3 | 4.1 | 4 | 2.3 | P = 0.1828 |
| **t127** | 1 | 1 | 3 | 4.1 | 4 | 2.3 | P = 0.1828 |
| **t186** | 1 | 1 | 3 | 4.1 | 4 | 2.3 | P = 0.1828 |
| t4609 | 1 | 1 | 2 | 2.7 | 3 | 1.7 |  |
| t11656 | 1 | 1 | 2 | 2.7 | 3 | 1.7 |  |
| t786 | 3 | 3 | 0 | 0 | 3 | 1.7 |  |
| t2771 | 0 | 0 | 3 | 4.1 | 3 | 1.7 |  |
| t078 | 2 | 2 | 0 | 0 | 2 | 1.2 |  |
| t3092 | 1 | 1 | 1 | 1.4 | 2 | 1.2 |  |
| t1456 | 1 | 1 | 1 | 1.4 | 2 | 1.2 |  |
| t189 | 2 | 2 | 0 | 0 | 2 | 1.2 |  |
| t2393 | 0 | 0 | 2 | 2.7 | 2 | 1.2 |  |
| t1376 | 0 | 0 | 2 | 2.7 | 2 | 1.2 |  |
| t1236 | 0 | 0 | 2 | 2.7 | 2 | 1.2 |  |
| t5739 | 0 | 0 | 2 | 2.7 | 2 | 1.2 |  |
| t5187 | 0 | 0 | 2 | 2.7 | 2 | 1.2 |  |
| t12939 | 0 | 0 | 1 | 1.4 | 1 | 0.6 |  |
| t3662 | 1 | 1 | 0 | 0 | 1 | 0.6 |  |
| t10394 | 1 | 1 | 0 | 0 | 1 | 0.6 |  |
| t1476 | 1 | 1 | 0 | 0 | 1 | 0.6 |  |
| t2168 | 1 | 1 | 0 | 0 | 1 | 0.6 |  |
| t213 | 1 | 1 | 0 | 0 | 1 | 0.6 |  |
| t130 | 1 | 1 | 0 | 0 | 1 | 0.6 |  |
| t2029 | 0 | 0 | 1 | 1.4 | 1 | 0.6 |  |
| t10277 | 1 | 1 | 0 | 0 | 1 | 0.6 |  |
| t729 | 1 | 1 | 0 | 0 | 1 | 0.6 |  |
| t509 | 1 | 1 | 0 | 0 | 1 | 0.6 |  |
| t7662 | 1 | 1 | 0 | 0 | 1 | 0.6 |  |
| t2680 | 0 | 0 | 1 | 1.4 | 1 | 0.6 |  |
| t616 | 0 | 0 | 1 | 1.4 | 1 | 0.6 |  |
| t4523 | 1 | 1 | 0 | 0 | 1 | 0.6 |  |
| t951 | 0 | 0 | 1 | 1.4 | 1 | 0.6 |  |
| **Total** | **99** |  | **73** |  | **172** |  |  |

- The predominant *spa* types are depicted in bold font. RF denotes Relative Frequency (%)
