# Supplementary figures and images for: Nasopharyngeal carriage, spa types and antibiotic susceptibility profiles of Staphylococcus aureus from healthy children less than 5 years in Eastern Uganda
Source: BMC Infect Dis. 2019 Dec 2;19:1023. doi: 10.1186/s12879-019-4652-5 (PMC6889221; doi:10.1186/s12879-019-4652-5)

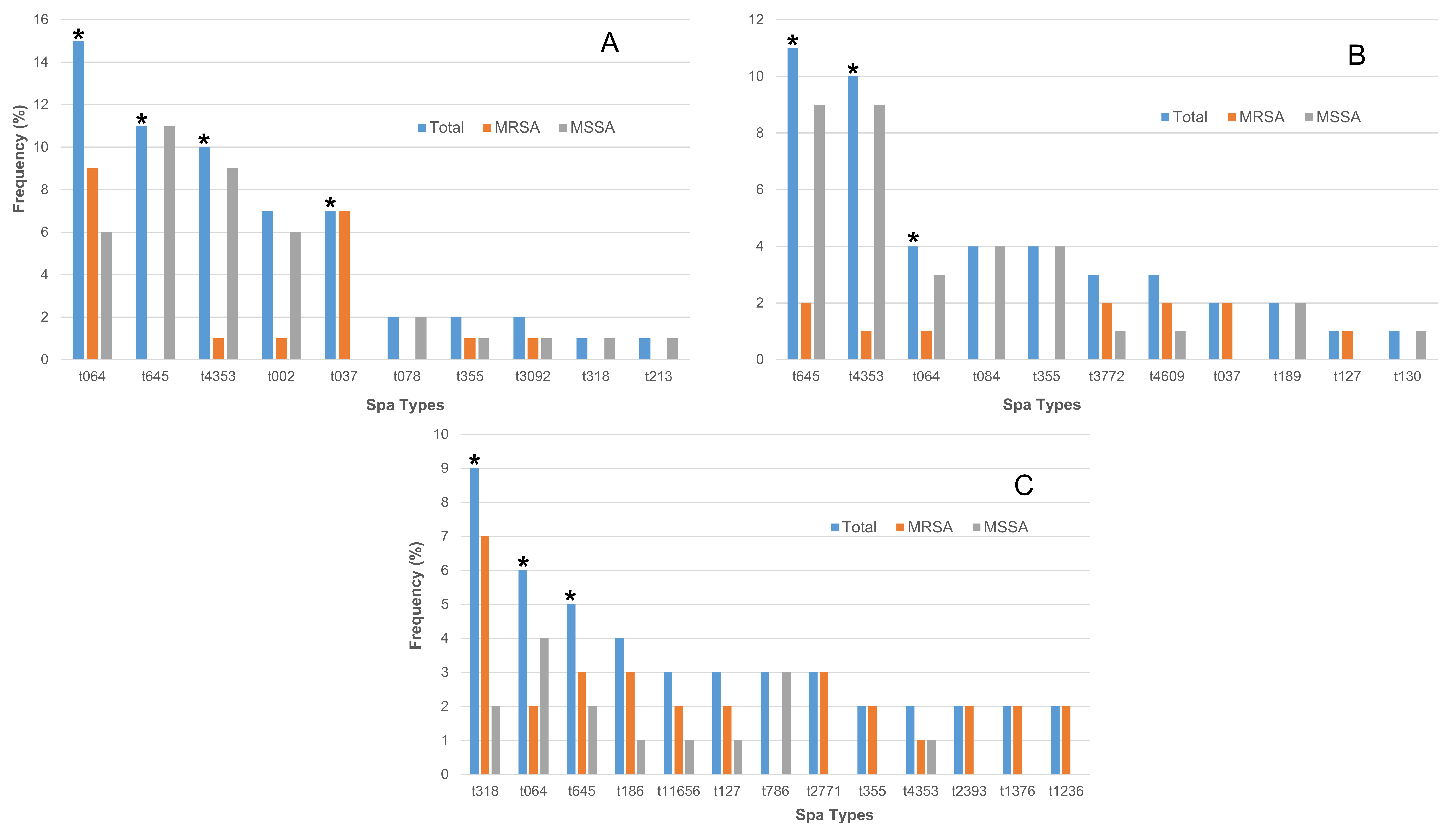

Supplement: Supplementary file 5 — Additional file 5: Figure S2. The most frequent spa types among S. aureus from the IMHDSS (panel A), Mulago Hospital (panel B) and rural western Uganda (panel C). Asterisks indicate frequencies for common spa types in Uganda and their association with either MRSA or MSSA. This analysis showed that MRSA infections in Uganda are more likely to be associated with spa types t064, t037, t645 and t318. [file 12879_2019_4652_MOESM5_ESM.tiff]
